# Supplementary material for: Comparative multiomics analysis of cell physiological state after culture in a basket bioreactor
Source: Sci Rep. 2022 Nov 23;12:20161. doi: 10.1038/s41598-022-24687-4 (PMC9686226; doi:10.1038/s41598-022-24687-4)
Supplement: Supplementary file 1 — Supplementary Information 1. [file 41598_2022_24687_MOESM1_ESM.zip › raw data/Proteomic raw data/6.KEGGenrichment/EG.vs.CG/ALL/pathway/src/00220.html]

map00220


- K00814, K14260, K14272, 2.6.1.2, R00258"

- Up regulated proteins

A0A0D9QZS1(Description:Alanine transaminase;Gene:GPT2;log2FC:1.4397)

Close
